# Titanium Surface Priming with Phase-Transited Lysozyme to Establish a Silver Nanoparticle-Loaded Chitosan/Hyaluronic Acid Antibacterial Multilayer via Layer-by-Layer Self-Assembly

**DOI:** 10.1371/journal.pone.0146957

**Published:** 2016-01-19

**Authors:** Xue Zhong, Yunjia Song, Peng Yang, Yao Wang, Shaoyun Jiang, Xu Zhang, Changyi Li

**Affiliations:** 1 School of Dentistry, Hospital of Stomatology, Tianjin Medical University, Tianjin, PR China; 2 Key Laboratory of Applied Surface and Colloid Chemistry, Ministry of Education, College of Chemistry and Chemical Engineering, Shaanxi Normal University, Xi’an, PR China; VIT University, INDIA

## Abstract

**Objectives:**

The formation of biofilm around implants, which is induced by immediate bacterial colonization after installation, is the primary cause of post-operation infection. Initial surface modification is usually required to incorporate antibacterial agents on titanium (Ti) surfaces to inhibit biofilm formation. However, simple and effective priming methods are still lacking for the development of an initial functional layer as a base for subsequent coatings on titanium surfaces. The purpose of our work was to establish a novel initial layer on Ti surfaces using phase-transited lysozyme (PTL), on which multilayer coatings can incorporate silver nanoparticles (AgNP) using chitosan (CS) and hyaluronic acid (HA) via a layer-by-layer (LbL) self-assembly technique.

**Methods:**

In this study, the surfaces of Ti substrates were primed by dipping into a mixture of lysozyme and tris(2-carboxyethyl)phosphine (TCEP) to obtain PTL-functionalized Ti substrates. The subsequent alternating coatings of HA and chitosan loaded with AgNP onto the precursor layer of PTL were carried out via LbL self-assembly to construct multilayer coatings on Ti substrates.

**Results:**

The results of SEM and XPS indicated that the necklace-like PTL and self-assembled multilayer were successfully immobilized on the Ti substrates. The multilayer coatings loaded with AgNP can kill planktonic and adherent bacteria to 100% during the first 4 days. The antibacterial efficacy of the samples against planktonic and adherent bacteria achieved 65%-90% after 14 days. The sustained release of Ag over 14 days can prevent bacterial invasion until mucosa healing. Although the AgNP-containing structure showed some cytotoxicity, the toxicity can be reduced by controlling the Ag release rate and concentration.

**Conclusions:**

The PTL priming method provides a promising strategy for fabricating long-term antibacterial multilayer coatings on titanium surfaces via the LbL self-assembly technique, which is effective in preventing implant-associated infections in the early stage.

## Introduction

Titanium (Ti) and its alloys are currently considered to be the most widely used biomaterials for dental implants because of their superior biocompatibility and excellent physicochemical properties [1]. However, implant-associated infection remains one of the most perilous complications of these procedures, leading to the failure of implant surgery along with psychological trauma and economic burden [2]. Recent studies have estimated that 65% of nosocomial infections are associated with biofilm, which has an enormous impact on medical therapies [3]. Peri-implantitis and peri-implant mucositis are generally difficult to manage owing to the long duration of antibiotic therapy and repeated surgical procedures [4]. The formation of biofilm on the surfaces of implants following by bacteria adhesion is the primary cause of infections of the mucosa and bone adjacent to the implant [5]. The process of biofilm formation involves unicellular organisms coming together to form a contiguous community encompassed in an exopolysaccharide matrix [6]. Thus, the biofilm makes the bacteria more invasive and competitive against the host defenses and presents difficulties for antibacterial treatments [7]. Therefore, establishing long-term antibacterial coatings on the surfaces of titanium implants to inhibit biofilm formation is of prime importance in the prevention of implant-associated infections.

Currently, the incorporation of antibacterial drugs into the coatings of dental implant surfaces has attracted increasing attention and is considered an effective strategy to prevent bacterial biofilm formation. Before loading antibacterial agents, it is essential to pretreat the implant surfaces using physical and chemical methods. Physical surface modification involves [8] physical vapor deposition, ion beam implantation and lithographic techniques; chemical methods are considered the most popular and efficient ways to modify implant surfaces and include acid etching, peroxidation, alkali treatment, anodic oxidation, incorporation of functional molecules via covalent crosslinking, chemical vapor deposition and hydrothermal modification [8]. However, these methods are inconvenient for application due to the involvement of complicated priming procedures, hazardous chemical substances and large-scale manufacturing equipment. Thus, a simple priming method for applying antibacterial coatings onto Ti surfaces is needed.

A novel phase-transited lysozyme (PTL) has recently been applied in surface functionalization. Compared with other traditional surface pretreatment techniques, priming pristine titanium surfaces with PTL is a simple, rapid, low-cost and green process for surface functionalization. The phase-transited lysozyme can be stably immobilized on a variety of substrates regardless of the substrate type, by the formation of an amyloid-like microfiber network based on the β-sheet transition found in lysozyme microfibers, which enables a robust adhesion to Ti surfaces [9]. This method is a one-step modification achieved by soaking the titanium surface in lysozyme transition buffer. In fact, priming with PTL places an initial layer of positive charges on the Ti surfaces for the fabrication of layer-by-layer self-assembly, on which the simple but robust immobilization of a series of functional building blocks can be accomplished through straightforward electrostatic interaction [9]. Consequently, it is possible to incorporate antibacterial agents into coatings on implants simply based on PTL pretreatment combined with this layer-by-layer self-assembly technique. Moreover, the PTL also has promising applications in surface modification of biomaterials for maxillofacial surgery, prosthodontics and orthopedics.

Recently, the efficacy of antimicrobial agents and the corresponding antibiotic resistance to these agents are still challenges to maintain long-term anti-infection treatments. Currently, silver nanoparticles, as strong antimicrobial agents, have attracted growing interest. In contrast with conventional antibiotics, silver nanoparticles have advantages such as strong antibacterial efficacy, a broad antibacterial spectrum that includes antibiotic-resistant bacteria and non-cytotoxicity in moderate doses [10, 11]. Furthermore, to inhibit biofilm formation, a composite structure must be fabricated on the Ti surfaces for sustained-release of Ag and long-term antibacterial activity. Layer-by-layer (LbL) self-assembly is a well-established versatile approach that fabricates a multilayer structure on Ti surfaces by depositing alternating layers of oppositely charged polyelectrolytes [12]. Multilayers composed of positively charged chitosan (CS) and negatively charged hyaluronic acid (HA) via LbL self-assembly are commonly used for drug delivery and release control due to their desirable biocompatibility [13, 14]. Moreover, chitosan also serves as a dispersant for silver nanoparticles [15]. Thus, in this study we applied the layer-by-layer (LbL) self-assembly technique to fabricate a silver nanoparticle-containing multilayer coating on PTL-primed Ti surfaces.

The aim of our study was to establish a novel initial layer on Ti surfaces with phase-transited lysozyme (PTL), based on which multilayer coatings with silver nanoparticles incorporated would be fabricated using chitosan (CS) and hyaluronic acid (HA) via the LbL self-assembly technique. We hypothesized that the silver nanoparticles-containing multilayer coating on the PTL-primed Ti surfaces would exhibit relatively long-term antibacterial efficacy and show favorable biocompatibility.

## Materials and Methods

### Materials

Pure titanium foils of 1 mm thickness and 12 mm diameter were purchased from Baoji Noble Metal Co., Ltd. (Shanxi, China). Chitosan ([2-anino-2-deoxy-(1–4)-β-D-glucopyranosel]), with a molecular mass of 400,000 Da and deacetylation degree of 100%, was purchased from Fluka. Sodium hyaluronate (1 g) and silver nitrate (25 g, AR, ≥99.8%) were purchased from SangonCo., Ltd. (Shanghai, China). *Staphylococcus aureus* (*S*. *aureus*, ATCC 25923) was obtained from China General Microbiological Culture Collection Centre. Lysozyme (2 mg/mL) dissolved in HEPES buffer (pH 7.4) and tris(2-carboxyethyl)phosphine (TCEP) (50 mM) were obtained from Shaanxi Normal University.

### Specimen preparation

The pure Ti foils were polished by SiC sandpaper of No.100, 240, 400, 600, 800 and 1000 grit in turn. Then, the foils were ultrasonically washed with acetone, ethanol and deionized water sequentially. Last, the Ti foils were sterilized in an autoclave at 120°C for 1 h for the *in vitro* experiments.

To functionalize the surface of the Ti discs, the samples were first dipped into a mixture of lysozyme and TCEP (1:1 in volume) and then incubated in a moist environment for 2 hours. These Ti discs were then washed with ultrapure water to remove residual impurities.

### Synthesis of silver nanoparticles

A chitosan solution was first prepared by dissolving 0.1% (w/v) chitosan in a 1% (v/v) acetic acid solution under stirring (1300 r/min). Next, silver nitrate powder was dissolved in the chitosan solution under stirring (1300 r/min) to obtain four concentrations (10, 20, 50 and 100 mM) of an AgNO_3_ solution. Subsequently, ascorbic acid (0.01 M) was added to the AgNO_3_ solution drop by drop with a pipette under constant stirring. All the above experiments were carried out at room temperature.

### Fabrication of multilayer coatings on the surface of PTL-primed Ti substrates

After the precursor layer was established, the substrate was sequentially treated with HA (1 mg/mL in 0.2 M sodium acetate buffer), washed with sodium acetate buffer, and then covered by CS/Ag nanoparticles. HA and CS/Ag were each defined as one monolayer and HA-CS/Ag was defined as one bilayer. These discs were denoted by Ti-PTL-HA-CS/Ag discs and were one typical cycle of multilayer construction. The immersion cycle was repeated three times until the desired multilayer coating was obtained (HA-CS/Ag-HA-CS/Ag-HA-CS/Ag). Finally, these samples were stored in a constant humidity chamber at 50±5% relative humidity before follow-up experiments.

**Fig 1:**
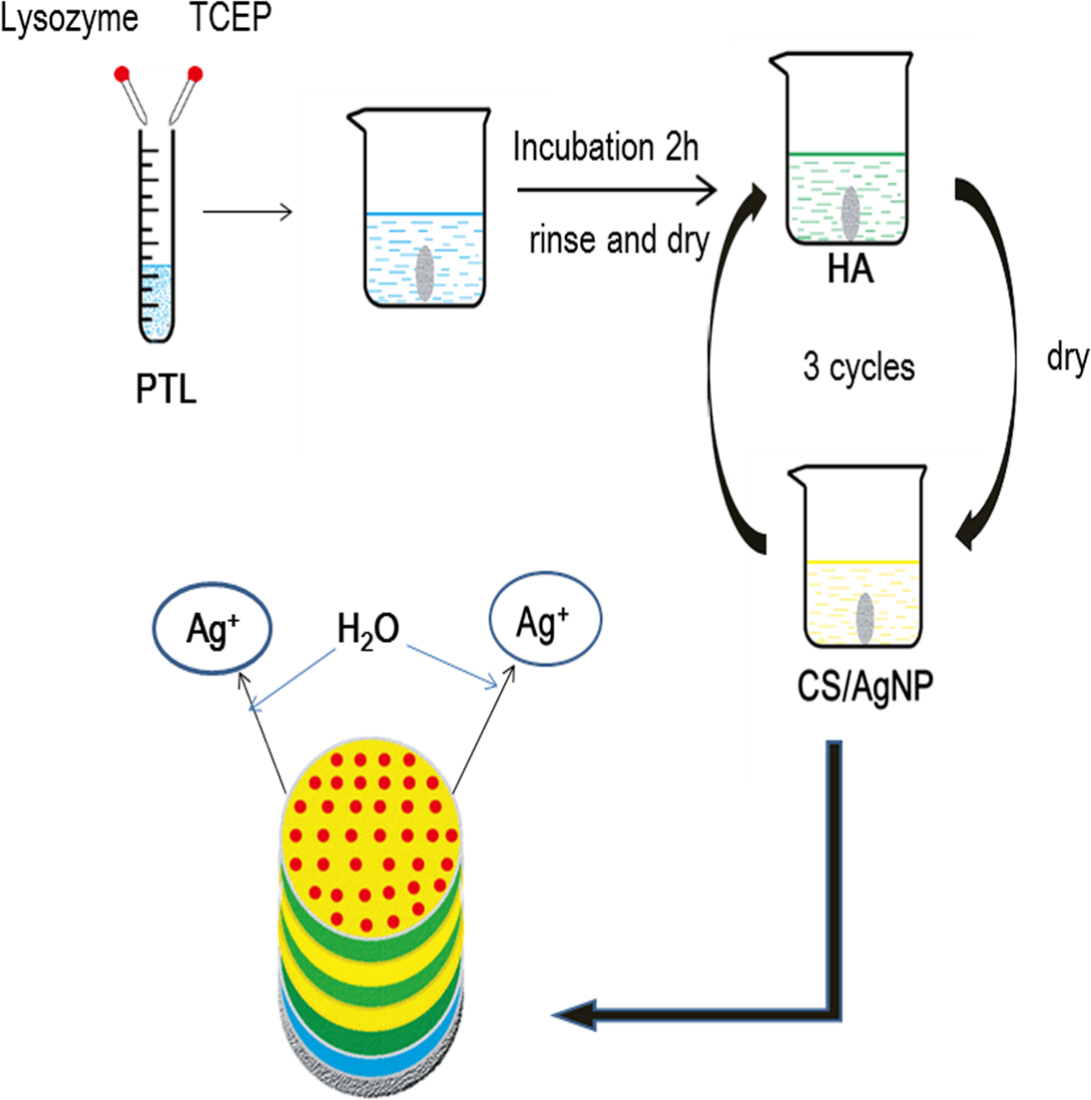
Schematic diagram: the process of fabricating the multilayer coatings on the PTL-primed Ti surface and the mechanism of antibacterial ability of this sample under aqueous conditions. As water contacts the surface, the nanoparticles are oxidized to release Ag^+^.

The whole process is shown in Fig 1.

### Release of silver incorporated into the composite *in vitro*

The amount of silver released from the samples was monitored in pH 7.4 phosphate buffered saline (PBS). The samples were immersed in 10 ml of PBS at 37°C in a oscillator (60 rpm/min) for 1 day in the dark, taken out, and then immersed again in 10 ml of fresh PBS. At different sampling intervals (1, 4, 7and 14 days), the supernatant was sampled for analysis. The PBS supernatant containing released Ag was analyzed by inductively coupled plasma atomic emission spectrometry (ICP-AES, Varian 725-ES, US).

### Surface characterization

X-ray photoelectron spectroscopy (XPS, AXIS His, Kratos Analytical Ltd., UK) was used to identify the chemical constituents of pristine and variously modified Ti surfaces. After immersed in 5 ml of PBS solution for 7days, the sample of Ti-PTL-HA-CS was determined by XPS to identify the chemical composition.

The contact angles of deionized water on the pristine and modified Ti surfaces were measured by the sessile drop method in a goniometer equipped with the drop-shape analysis system (JC2000D1, Micaren, China) at room temperature. Each Ti disc was measured three times to calculate the mean values of the contact angles.

The surface morphology of the pristine and decorated Ti was characterized by field-emission scanning electron microscopy (FE-SEM, JSM-5600LV, JEOL, Japan) with a beam voltage of 15kV. All the samples were sputter-coated by gold before SEM observation except for the Ti discs modified with CS/AgNP. The chemical composition of the surface of the CS/AgNP discs was identified by energy dispersive X-ray detector (EDX, Japan).

The size and morphology of the Ag nanoparticles was observed by transmission electron microscopy (TEM, Philips CM20).

### Antibacterial assay

*Staphylococcus aureus* (*S*. *aureus*, ATCC 25923) was cultivated in a beef extract-peptone (BEP) medium. After an overnight culture at 37°C, the bacterial suspension was adjusted to a concentration of 10^5^ CFU/ml for the antibacterial assay. Ti discs were put into sterilized 24-well plates filled with a bacterial suspension (1 ml per well) and cultured at 37°C in an incubator. At different intervals (1, 3, 5, 7 and 14 days), the bacterial suspension was sampled and then the detached biofilm clumps were disaggregated by sonication. The viable planktonic bacteria were counted using serial dilutions and the spread plate method. Next, the Ti discs were taken out, gently rinsed with PBS to eliminate non-attached bacteria and then underwent ultrasonic treatment at 40 W for 5 min in a new 24-well plate filled with 1 ml of BEP per well, followed by sampling the bacterial suspension to count the viable bacteria adhered to the Ti discs. During the incubation period, the former medium was replaced by a new culture medium of bacterial suspension every day.

The antibacterial efficacy of samples against planktonic bacteria and adhered bacteria were determined by the following formula: R = (B-A)/B×100%. A is the number of viable bacteria in the culture medium with a modified/pristine Ti disc or on a modified Ti disc. B is the number of viable bacteria in the culture medium without a Ti disc or on a pristine Ti disc.

Fluorescence staining for live and dead bacteria was used to characterize the viability of adherent bacteria on the samples. *S*. *aureus* was seeded on the surfaces of the Ti discs in a 24-well plate, as with the incubation, for 7 days, as previously described. The bacterial medium was refreshed daily, and after 7 days, the samples were rinsed with PBS to remove non-adherent bacteria. Then, the bacterial cells were stained with acridine orange and ethidium bromide for 15 min in the dark before observation by confocal laser scanning microscopy (CLSM) (TCS SP5, Leica, Germany).

### Cell culture

MC3T3-E1 murine preosteoblasts (Type Culture Collection of the Chinese Academy of Sciences, Shanghai, China) were used for cytotoxicity tests. Cells were cultured in DMEM medium (Gibco, Carlsbad, CA) containing 10% fetal bovine serum (FBS) (Gibco) and 3% penicillin/streptomycin (Gibco) at 37°C in a humidified atmosphere of 5% CO_2_.

### Lactate dehydrogenase activity assay

The cytotoxicity of AgNP to MC3T3 cells can be assessed by the activity of lactate dehydrogenase (LDH, Sigma-Aldrich) released by the cells in the culture media. The MC3T3 cells were seeded on each specimen in a 24-well plate at a density of 2×10^4^ cells per well. After incubation for 1 and 4 days, the culture media were sampled and centrifuged, and then, the supernatant was used for the LDH activity assay. LDH activity was determined by the absorbance value of optical density (OD) at a 450-nm wavelength according to the manufacturer’s instructions.

### Alkaline phosphatase activity

One milliliter of the MC3T3 cell suspension was seeded on each specimen in a 24-well plate at a density of 1×10^5^ cells per well. The cells were cultured for 7 days, then washed with PBS and lysed in 0.1 vol% Triton X-100 through the standard freeze-thaw cycles. The alkaline phosphatase (ALP) activity in the cell suspension was determined by the absorbance value at 520 nm. The ALP activity was normalized to the total protein content which was analyzed by the MicroBCA protein assay kit.

### Cell viability assay

To evaluate the *in vitro* cell viability of the MC3T3 cells on each sample (2×10^4^ cells/well), a cell counting kit-8 assay (CCK-8, Dojindo, Japan) was performed according to the manufacturer’s instruction. In brief, after incubation for 3 days, the cells were collected and centrifuged before they were placed in a 96-well plate with 100 μl fresh medium per well. Next, a 25-μl CCK-8 solution was added to each well of the 96-well plate and kept at 37°C, 5% CO_2_. After two hours, the production of formazan by viable cells was assessed through the absorbance value of supernatant optical density (OD), which was measured with a microplate reader (model 680, Bio-Rad, CA) at a 450-nm wavelength.

### Statistical analysis

Each test was repeated three times, and the results were expressed as the means ± standard deviations. The data were tested for homogeneity and then assessed statistically using one-way ANOVA and a Student-Newman-Keuls (SNK) post hoc test. P < 0.05 was considered significant and p < 0.01 was considered highly significant.

## Results

### Surface characterization

**Fig 2:**
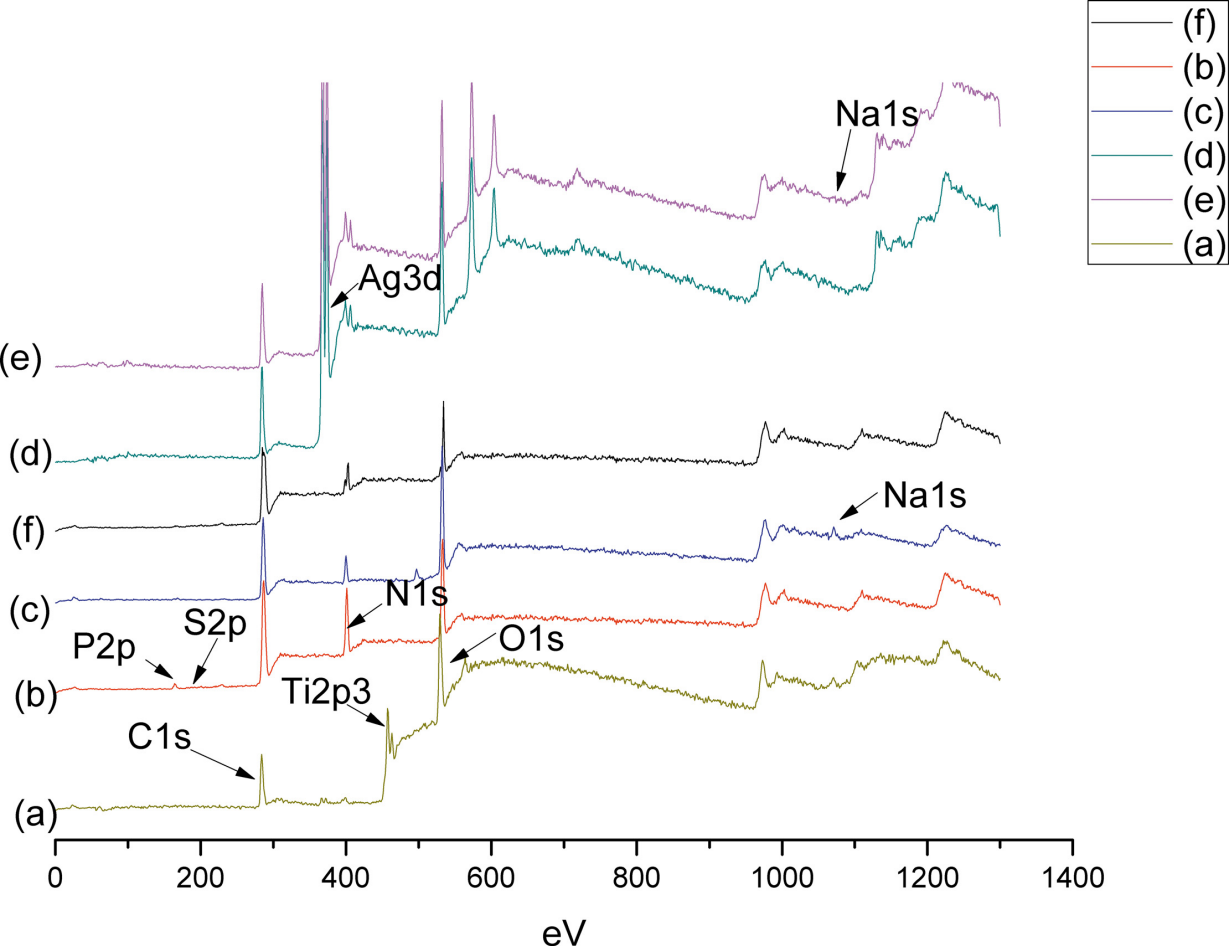
XPS wide-scan spectra of (a) pristine Ti, (b) PTL treated Ti (Ti-PTL), (c) Ti-PTL-HA, (d) Ti-PTL-HA-CS/Ag, (e) Ti-PTL-HA-CS/Ag–HA, (f) Ti-PTL-HA-CS after immersion for 7days.

The chemical constituents of the surface of the pristine and modified Ti discs in various stages of LBL self-assembly were analyzed by XPS. The XPS spectra of surface elemental compositions after calibrating peak positions, using C 1s as an internal reference at 284.8 eV, are shown in Fig 2 and Table 1. The wide-scan spectrum of the pristine Ti (Fig 2A) shows that the chief components include C 1s, Ti 2p3 (458.5 eV), O 1s (530 eV) and N 1s (399 eV). The distinctive peaks of P 2p (132 eV) and S 2p (164 eV) originated from TCEP, and the disappearance of the Ti 2p3 peak indicates successful anchoring of PTL to the Ti substrate (Fig 2B**)**, which is also supported by the quantitative analysis of the XPS results (Table 1). As shown in Fig 2C and Table 1, Na content originated from hyaluronate indicates that hyaluronic acid (HA) was immobilized on the PTL-primed Ti surface. Upon the addition of CS/AgNP to the layer of HA, the distinctive Ag content appears due to the Ag-containing in chitosan. Fig 2F shows the moderate decrease in height of N1s peak and the absence of P2p and S2p peak of the sample of Ti-PTL-HA-CS after immersion for 7days.

**Table 1:** Elemental composition at the surface of various Ti discs with different treatments as determined by XPS.

	C%	O%	N%	Ti%	Na%	S%	Ag%	P%
Ti	47.2±0.2	38.2±0.3	4.4±0.1	10.2±0.03	0	0	0	0
Ti-PTL	62.7±0.3	18.3±0.2	18.2±0.1	0	0	0.7±0.1	0	0.1±0.03
Ti-PTL-HA	60.2±0.4	28.8±0.3	9.0±0.2	0	2.0±0.02	0	0	0
Ti-PTL-HA-CS/Ag	49.2±0.2	26.5±0.1	10.6±0.3	0	0	0	13.8±0.2	0
Ti-PTL-HA-CS/Ag-HA	46.8±0.1	28.2±0.1	11.9±0.2	0	0.2±0.02	0	12.9±0.2	0

The surface hydrophilicity of pristine and modified Ti discs was also investigated. As depicted in Table 2, the water contact angle on the Ti surface coated with the LbL self-assembled multilayer containing CS/AgNP is sharply decreased from 77.2°±1.5° to 49.0°±1.3°. The surface of the pristine Ti discs was more hydrophobic, while the surface of the modified Ti discs was hydrophilic.

**Table 2:** Contact angle (mean± SD) of the various samples.

	Contact angle (°)
Pristine Ti	77.2±1.5^a^
Ti-PTL	72.5±1.5^a^
Ti-PTL-HA-CS/Ag	64.5±2.6^b^
LbL-CS/Ag	49.0±1.4^b^

In the same column, values with different lowercase letter superscripts mean significant differences (p<0.05) compared with pristine Ti.

**Fig 3:**
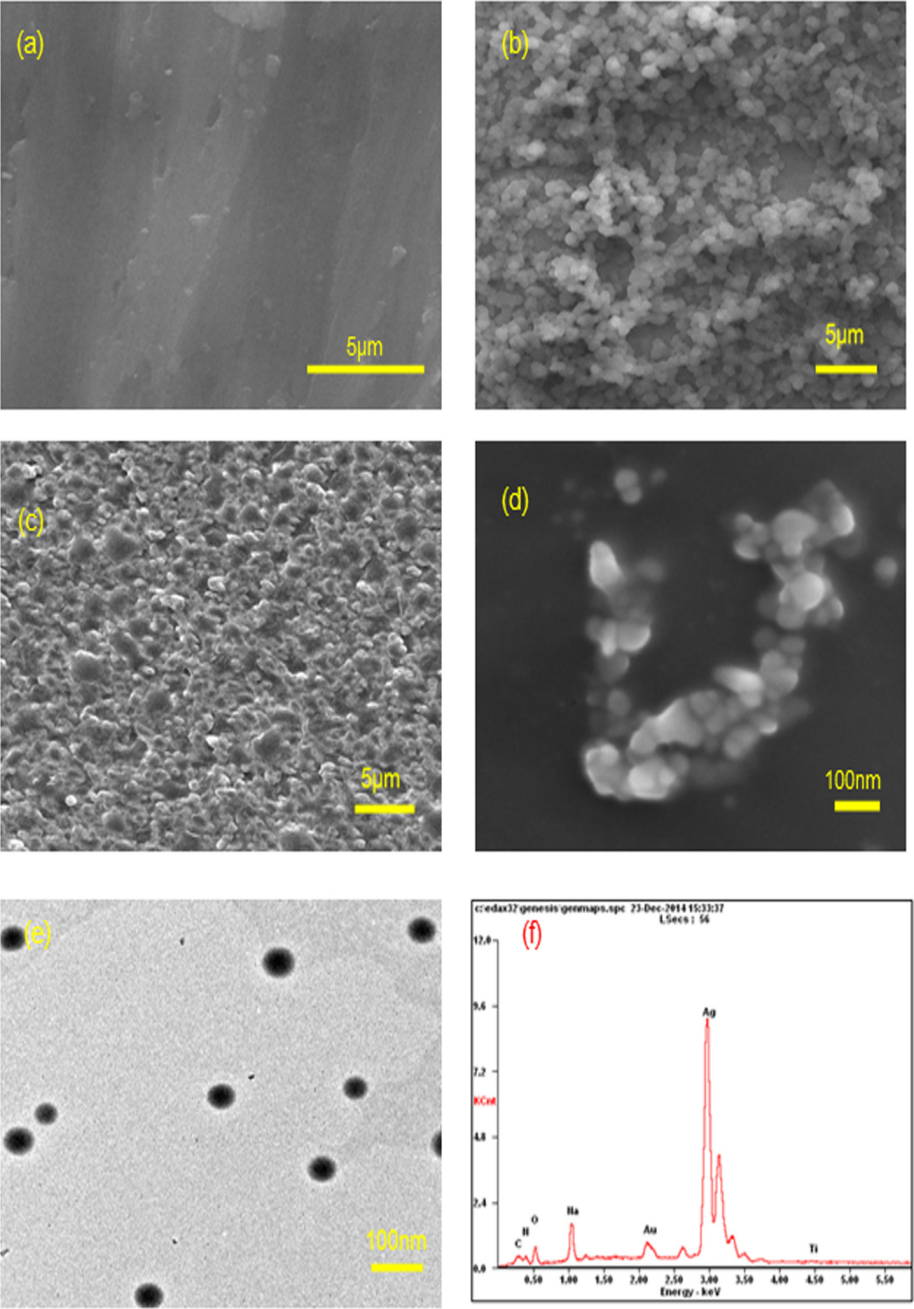
SEM images of surface morphology of Ti discs: (a) pristine Ti, (b) Ti-PTL, (c) Ti-PTL-HA, (d) Ti-PTL-HA-CS/Ag10. (e) TEM image of CS/Ag (10mM). (f) EDX image of the sample of Ti-PTL-HA-CS/Ag.

SEM results (Fig 3A–3D) show the surface morphology of a pristine Ti disc, PTL primed Ti disc, HA-coated PTL-Ti and multilayer of HA and CS/AgNP coated Ti disc. The SEM image (Fig 3B) reveals that the PTL has necklace-like fibers with a diameter of 0.5–1μm which is in good agreement with a previous report [9]. The EDX pattern (Fig 3E) also verifies that the chemical composition of self-assembled multilayer loaded onto the Ti surface is silver. The morphology of the composite CS/AgNP (10 mM) is exhibited in the TEM results (Fig 3F). The shape of the silver nanoparticles was a relatively uniform sphere averaging approximately 30 nm in size.

### Release of Ag from Ag nanoparticles-loaded Ti discs

**Fig 4:**
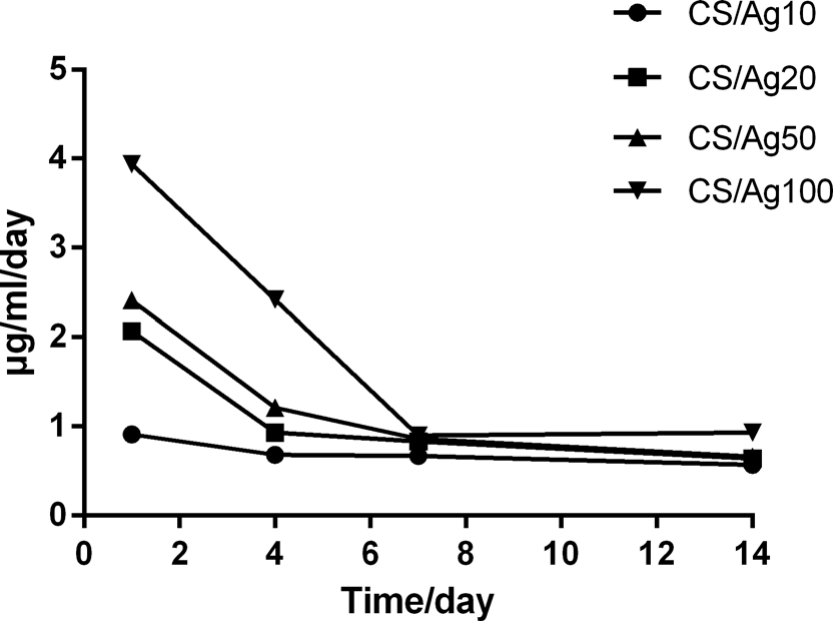
Non-cumulative silver release profile in PBS.

As shown in Fig 4, the Ag released from the samples in PBS exhibits an initial burst effect on the first day. The amount of Ag released at the different intervals (1, 4, 7 and 14 days) follows the order of CS/Ag100 > CS/Ag50 > CS/Ag20 > CS/Ag10. Initially, relatively large amounts of Ag were released into the PBS, with CS/Ag100 leaching the most, and the amount of released Ag decreased gradually with immersion time and tended to be stable after 7 days. After two weeks, the average concentration of released Ag was 0.70±0.14 μg/ml.

### Inhibition of biofilm formation

**Fig 5:**
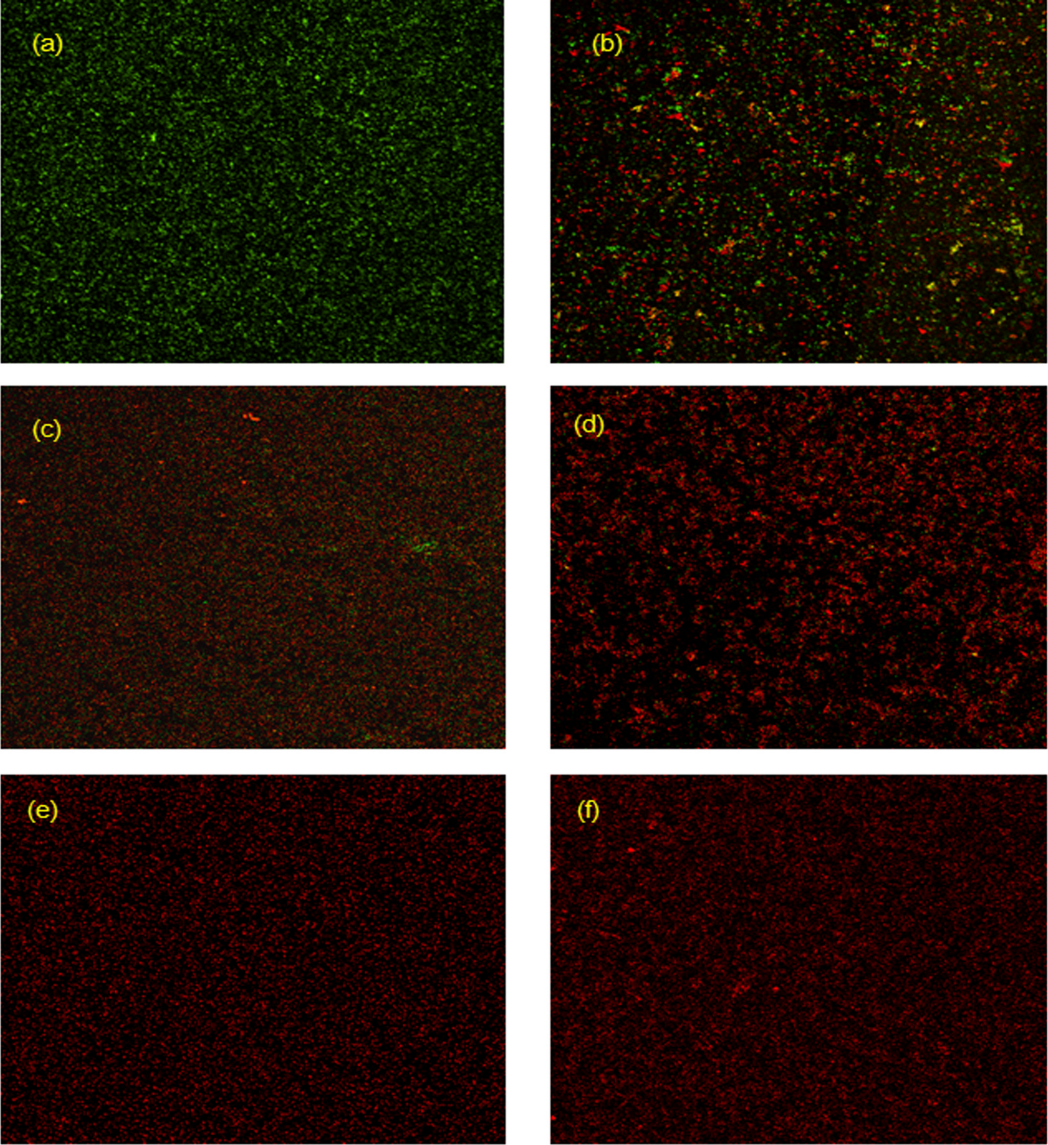
CLSM fluorescence microscopy images of (a) pristine Ti, (b) Ti-PTL, (c) LbL-CS/Ag10, (d) LbL-CS/Ag20, (e) LbL-CS/Ag50, (f) LbL-CS/Ag100 showing viability of the bacteria on samples after 7 days.

The antimicrobial ability of CS/Ag-decorated Ti discs was investigated by fluorescence staining. Fig 5 shows the CLSM images of adherent bacteria on the pristine and modified Ti discs after 7 days. The Ti discs were incubated in the culture media with repeated bacteria invasion every day. The fluorescence microscopy images of CLSM showed more dead *S*. *aureus* cells on the surfaces of the CS/Ag samples with red color. In addition, viable bacteria with green color were observed on the surfaces of pristine Ti and PTL-primed Ti. In significant contrast, nearly no viable bacterial cells could be found on the CS/Ag modified Ti surfaces.

**Fig 6:**
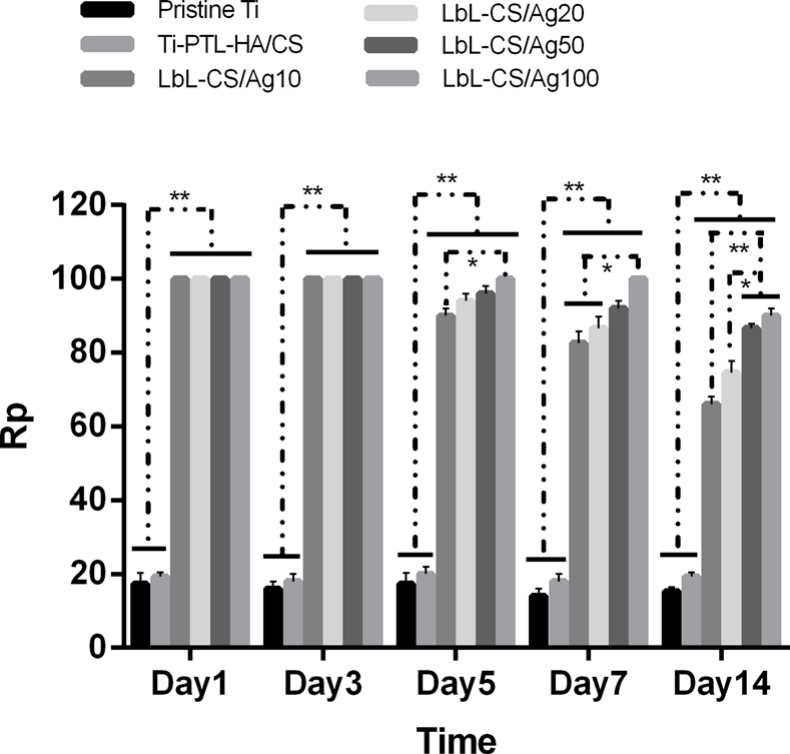
Antibacterial rates against planktonic bacteria in medium (Rp). The antibacterial assays data are expressed as means±standard deviations (n = 3). One-way ANOVA followed by SNK post hoc test is utilized to determine the level of significance. *p<0.05 and **p<0.01.

**Fig 7:**
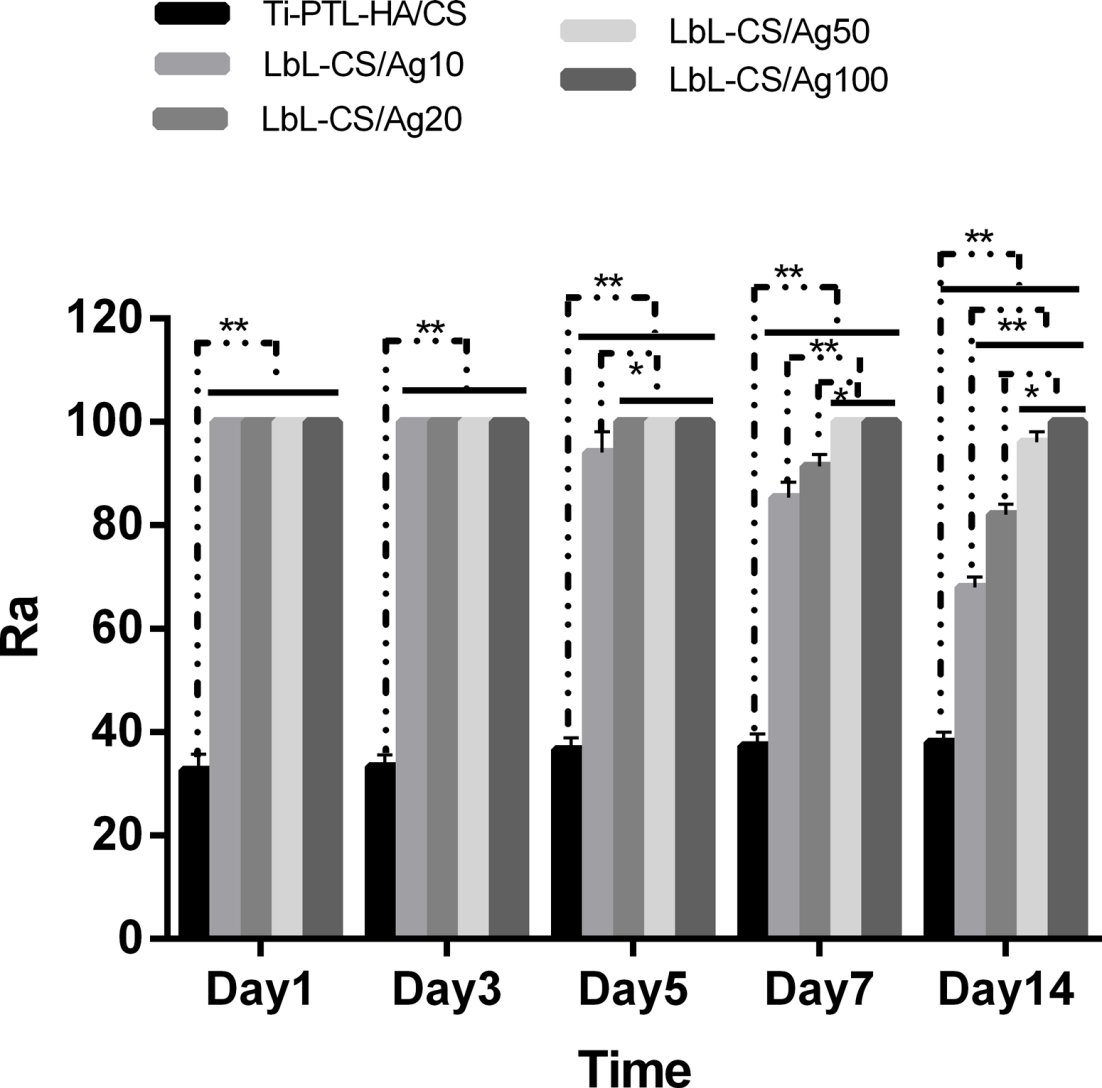
Antibacterial rates against adherent bacteria on the specimens (Ra). The antibacterial assays data are expressed as means±standard deviations (n = 3). One-way ANOVA followed by SNK post hoc test is utilized to determine the level of significance. *p<0.05 and **p<0.01.

The antibacterial efficacy of samples against planktonic bacteria in the medium (Rp) and adherent bacteria on the surfaces of samples (Ra) over 14 days were evaluated, as shown in Figs 6 and 7, respectively. The CS/Ag samples showed Rp values of approximately 100%, significantly higher than pristine Ti and Ti-PTL-HA/CS during the first 4 days. At the 5^th^ day, the Rp values of the CS/Ag samples decreased gradually, and those of the CS/Ag10 samples diminished more rapidly. After 7 days, the Rp values of the Ti-PTL-HA/CS and CS/Ag samples, particularly the CS/Ag100 samples, were significantly higher than those of pristine Ti samples. The modified Ti surfaces with silver incorporated were effective in preventing bacteria colonization on the Ti discs for 14 days as illustrated by Fig 7. Most of the CS/Ag samples showed Ra values of 100% without a significant decrease over 7 days. However, the CS/Ag10 samples exhibited a relatively sharp decrease after 14 days reaching a value of approximately 65%. The other three CS/Ag groups still showed Ra values of approximately 90% after 14 days.

### Cytotoxicity

**Fig 8:**
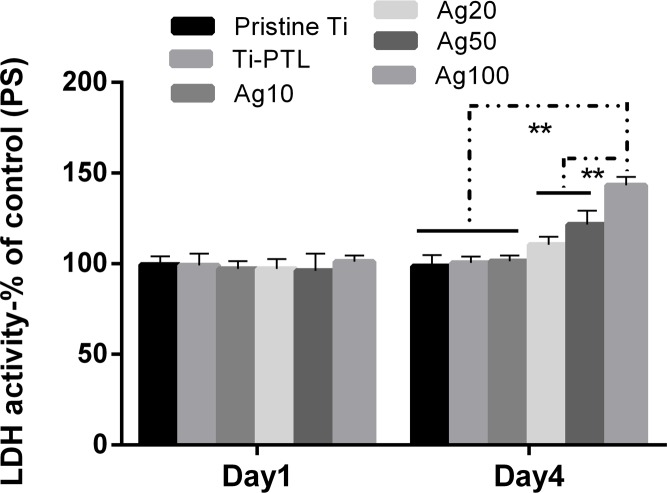
LDH activity in medium after culturing for 1 and 4 days on the specimens. The data are expressed as means±standard deviations (n = 3). One-way ANOVA followed by SNK post hoc test is utilized to determine the level of significance. *p<0.05 and **p<0.01.

The cytotoxicity results indicated by the LDH activity in the supernatants after 1 and 4 days of incubation are compared in Fig 8. After the first day, neither the Ti-PTL nor the CS/Ag samples showed an obvious enhancement in LDH activity. After culturing for 4 days, the CS/Ag20 and CS/Ag50 samples exhibited slightly higher LDH activity than pristine Ti, Ti-PTL and CS/Ag10, but the difference was statistically insignificant. However, higher LDH activity was observed in the CS/Ag100 sample. The CS/Ag samples exhibited cytotoxicity with the increase in the amount of incorporated Ag.

### Cell viability

**Fig 9:**
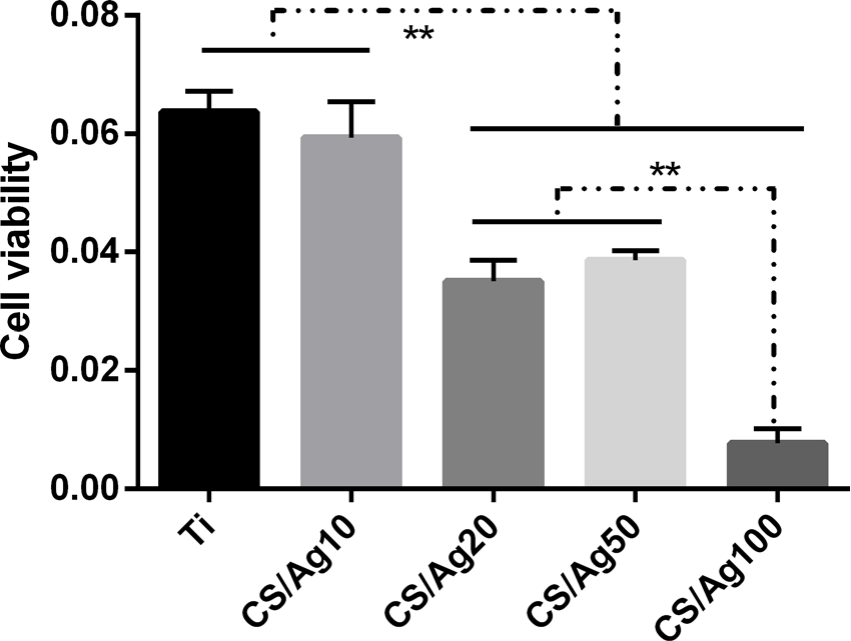
Cell viability of MC3T3 on the specimens after culturing for 3 days determined by CCK-8 assay. The data are expressed as means±standard deviations (n = 3). One-way ANOVA followed by SNK post hoc test is utilized to determine the level of significance. *p<0.05 and **p<0.01.

The cell viability of each sample was evaluated by a Cell Counting Kit 8 (CCK-8). As shown at Fig 9, the CS/Ag20, CS/Ag50 and CS/Ag100 samples show significant differences without the CS/Ag10 group. Moreover, the CS/Ag100 samples exhibited significantly lower cell viability than the other samples.

### Alkaline phosphatase activity

**Fig 10:**
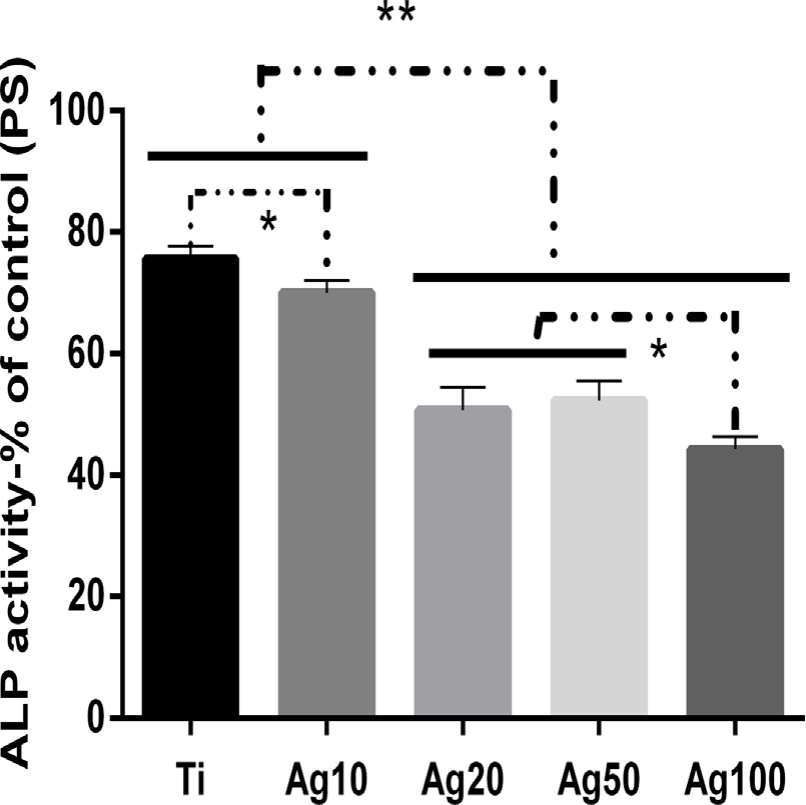
ALP activity of MC3T3 on the specimens after incubation for 7 days. The data are expressed as means±standard deviations (n = 3). One-way ANOVA followed by SNK post hoc test is utilized to determine the level of significance. *p<0.05 and **p<0.01.

The ALP activity assay after 7 days of culturing is shown in Fig 10. Compared with pristine Ti, the modified Ti discs with Ag incorporated had decreased ALP activity, particularly the CS/Ag100 samples, which exhibited dramatically lower ALP activity (20–35%).

## Discussion

In this study, we tried to develop a distinctive and simple pretreatment method: using phase-transited lysozyme (PTL) to modify the Ti surfaces. The initial layer of PTL on Ti surfaces would provide a base for antibacterial multilayer coatings established via a layer-by-layer self-assembly technique. Ideally, a broad spectrum of building blocks including small and macro-molecules, biomolecules and colloids, could be easily immobilized onto a PTL-primed substrate, regardless of substrate type [9]. XPS results (Fig 2B) demonstrated that the PTL was successfully immobilized onto the surface of Ti discs. It has been suggested that PTL is an extremely stable material and the adhesion strength of PTL is strong enough to endure ultrasonic vibration [9]. The PTL immobilized on substrates remains intact in various polar and non-polar organic solvents as well as acids, bases, inorganic salts, surfactants and oxidants, with the exception of guanidine solution (GndCl) [9]. The adhesion feature of PTL originates from amyloids found in the lysozyme fibers, which have commonly been used as proteinaceous underwater adhesives for bioadhesion [9, 16]. This mechanism may be ascribed to a complex sequence of events and the co-contributions of multi-scale molecular and structural amyloid bonds including osmotic pressure-driven solvent depletion force, hydrophobic interactions, physical entanglement and hydrogen bonding/electrostatic interactions [9]. In addition, compared with negatively charged dopamine, which is widely applied to prime surfaces for further modification [17], PTL contains polar functionalities such as amines and hydroxyls with mild positive charges over a broad pH range for robust immobilization of negatively charged functional building blocks on Ti substrates [9].

The formation of biofilm around dental implants is the essential factor in the evolution and persistence of infection [18]. Moreover, the surgical trauma of implantation can disturb the host defense system which facilitates bacterial colonization [19]. Thus, it is absolutely necessary to develop effective strategies to prevent implant-associated infections. Although antibacterial coatings loaded onto Ti surfaces have attracted considerable attention due to the effective inhibition of biofilm formation, relatively long-term antibacterial effect is crucial to protect biomedical implants against the constant risk of infection before mucosal healing. In contrast with monolayer antibacterial coatings, multilayer coatings on a Ti surface constructed by layer-by-layer (LbL) self-assembly technique can enhance loading capacity and control the release of antibacterial agents to achieve a long-term antibacterial effect [20].

In the present work, multilayer coatings on PTL-primed Ti surfaces were fabricated by alternate adsorptions of polyanions (hyaluronic acid) and polycations (chitosan) through electrostatic interaction based on a LbL self-assembly technique. XPS results indicated that the self-assembled multilayer of chitosan and hyaluronic acid was successfully coated onto the surface of Ti discs (Fig 2D and Table 1). Moreover, Fig 2F indicated the LbL method was stable to stay for the intended duration. The chitosan layers were loaded with Ag nanoparticles as antibacterial agents. In present study, the Ag nanoparticles were synthesized in a chitosan medium with the addition of the reducing agent VC. The linear macromolecules of chitosan will form tridimensional gridding structures, providing host spaces for silver ions [21]. Thus, in the chitosan medium, the controlled synthesis of Ag nanoparticles with controlled size, morphology and dispersity can be accomplished when these silver ions are reduced in situ to form nanoparticles as shown in Fig 3E, producing complexes of chitosan and Ag nanoparticles (CS/AgNP). Moreover, the complexes of CS/AgNP still remained positively charged and thus could be directly adsorbed onto the layer of HA (Figs 2D and 3D). This one-step fabrication of complexes of CS/AgNP is a simple and convenient method to load an antibacterial coating onto Ti surfaces via the LbL self-assembly technique.

In the present work, the multilayer coatings loaded with various concentration of AgNP showed effective antibacterial activity over a 14-day period (Figs 6 and 7). The results suggested that the antibacterial activity is enhanced with increasing concentrations of AgNP. The planktonic bacteria in the medium and the adherent bacteria on the surfaces of samples were almost eradicated by AgNP released from the self-assembled multilayer during the first 5 days, thus reducing the bacterial colonization of the surfaces of samples. The antimicrobial effect could predominantly be ascribed to the release of Ag^+^ from AgNP, which has the ability to inhibit bacterial DNA replication, interrupt bacteria cellular processes and induce reactive oxygen species (ROS) [22, 23]. ROS can increase the permeability of the bacterial membrane, causing bacteria to be more susceptible to antibacterial agents. In addition, unlike the relative hydrophobicity of pristine Ti, the hydrophilicity of Ti surfaces coated with a multilayer of HA and CS/AgNP (Table 2 and Fig 7) contributes to the reduction in bacterial cells [20]. The results of the antibacterial efficacy against adherent bacteria on the surfaces of samples demonstrate that the self-assembled multilayer of chitosan and hyaluronic acid loaded with AgNP can effectively inhibit biofilm formation on Ti surfaces over 14 days.

An adequate period of time and a suitable profile of drug release at an effective antibacterial concentration are necessary to inhibit biofilm formation before wound healing. Initially, a large amount of Ag was released from the self-assembled multilayer into the PBS, which was attributed to the outer most layer of chitosan loaded with AgNP, but Ag release gradually decreased with the increase in immersion time (Fig 5). The Ag release profile over 14 days observed in our study is advisable because a surgical wound will heal within 10–14 days, after which the constant release of Ag is not recommended due to cytotoxicity to the host cells. The initial burst of Ag release inhibits the immediate colonization of bacteria on the Ti surfaces after implant surgery. Next, sustained release of Ag is required to resist bacterial invasion from the outer edges of the wound until mucosal healing. In this study, the four concentration levels of AgNP were able to effectively kill planktonic and adherent bacteria over 14 days; the cytotoxicity of the four levels was also investigated to find a proper concentration for ensuring normal mucosal healing.

Concerning the toxicity of silver, it is generally considered that Ag is biocompatible at low concentrations and cytotoxic to host cells at high doses [24, 25]. This is related to a large release of Ag ions due to the high surface energy of smaller AgNP [22]. For biomedical implant applications, smaller Ag particle size does not always lead to better performance. In our work, in comparison to pristine Ti, the multilayer coatings with AgNP incorporated (10, 20, 50, 100 mM) still showed cytotoxicity against MC3T3 cells to a certain extent, as demonstrated by the LDH, ALP activity and cell viability assays (Figs 8–10). However, the samples of CS/Ag10 with the lowest concentration of AgNP showed better biocompatibility than other groups during the first 4 days according to the LDH activity assay results (Fig 8). The cytotoxicity of the samples may be attributed to the leaching of silver ions and their accumulation in the culture medium during the incubation period [22, 25, 26]. After 3 days of culturing without changing the culture medium, the cumulative concentration of silver ions led to cytotoxicity at day 4. However, recent studies indicate that biomaterials containing a proper amount of silver are biocompatible with osteoblasts [10, 11, 27, 28]. As a result, it is desirable that the bacteria can be killed without inducing cell cytotoxicity at a properly low concentration of silver ions. Ti surfaces coated with a HA-CS/AgNP multilayer via PTL-priming and the LbL self-assembly technique can exhibit good biocompatibility by controlling the release of silver. Therefore, the antibacterial and biocompatible surfaces of titanium modified with the HA-CS/AgNP multilayer have a property that prevents post-operation infection in the early stage of implantation, which will be further investigated through future *in vivo* experiments.

In conclusion, in contrast to other well-established methods, our surface-priming strategy provides an extremely facile, green and powerful approach to preparing Ti surfaces by using PTL coating, without time-consuming chemical synthesis and costly processing [9]. Therefore, the initial layer of PTL holds great potential for the fabrication of multilayers loaded with antibacterial agents, osteogenic growth factors, cytokines and/or other functional components on Ti surfaces via the LbL self-assembly technique, which could prevent implant associated infection and facilitate osseointegration in the early stage of implantation. Furthermore, the substrate-independent PTL can served as the base layer to fabricate coatings of silver nanoparticles on other medical devices such as catheters, wound dressing and bone cements[29].

## Supporting Information

S1 Text Minimal Dataset. (DOCX)

S2 Text Paper revised by AJE with Track Change. (DOCX)
