# Supplementary material for: Titanium Surface Priming with Phase-Transited Lysozyme to Establish a Silver Nanoparticle-Loaded Chitosan/Hyaluronic Acid Antibacterial Multilayer via Layer-by-Layer Self-Assembly
Source: PLoS One. 2016 Jan 19;11(1):e0146957. doi: 10.1371/journal.pone.0146957 (PMC4718720; doi:10.1371/journal.pone.0146957)
Supplement: S1 Text — (DOCX) [file pone.0146957.s001.docx]

| CCK-8 TEST | | | |  | |  | |  |  |
| --- | --- | --- | --- | --- | --- | --- | --- | --- | --- |
|  |  | | |  | |  | |  |  |
|  | Ti | | | CS/Ag10 | | CS/Ag20 | | CS/Ag50 | CS/Ag100 |
| OD | 0.064 | | | 0.054 | | 0.038 | | 0.04 | 0.005 |
|  | 0.06 | | | 0.066 | | 0.036 | | 0.037 | 0.01 |
|  | 0.067 | | | 0.058 | | 0.031 | | 0.039 | 0.008 |
| Contact angle(℃)  Ti | Ti-PTL | | | Ti-PTL-HA-CS/Ag | | LbL | |  |  |
| 79.3 | | 72.9 | 65.5 | | 49.3 | |  |  |  |
| 76.9 | | 74.1 | 67.1 | | 47.2 | |  |  |  |
| 75.6 | | 70.5 | 60.9 | | 50.5 | |  |  |  |
|  |  | | |  | |  | |  |  |
