# Supplementary material for: Titanium Surface Priming with Phase-Transited Lysozyme to Establish a Silver Nanoparticle-Loaded Chitosan/Hyaluronic Acid Antibacterial Multilayer via Layer-by-Layer Self-Assembly
Source: PLoS One. 2016 Jan 19;11(1):e0146957. doi: 10.1371/journal.pone.0146957 (PMC4718720; doi:10.1371/journal.pone.0146957)
Supplement: S2 Text — (DOCX) [file pone.0146957.s002.docx]

**Surface Priming of Titanium Substrates with Phase-transited Lysozyme to Establish a Silver Nanoparticle-loaded Chitosan/hyaluronic Acid Multilayer via Layer-by-layer Self-assembly for Inhibition of Biofilm Formation**

Xue Zhong ^1^, Yunjia Song^1^, Peng Yang ^2^, Yao Wang^1^, Shaoyun Jiang ^1^, Xu Zhang ^1,*^, Changyi Li ^1,*^

^1^ School of Dentistry, Hospital of Stomatology, Tianjin Medical University, 12 Observatory Road, Tianjin 300070, PR China

^2^ Key Laboratory of Applied Surface and Colloid Chemistry, Ministry of Education, College of Chemistry and Chemical Engineering, Shaanxi Normal University, Xi’an 710062, PR China

* Corresponding authors

Email: zhxden@gmail.com, zhangxu@tmu.edu.cn (Xu Zhang)

cli02@tmu.edu.cn (Changyi Li)

**Abstract**

Objectives: Bacterial colonization immediately after installation, which is induced by the formation of biofilm around implants, is the primary cause of post-operation infection. Initial surface modification is usually required to incorporate antibacterial agents on titanium surfaces to inhibit biofilm formation. However, simple and effective priming methods are still lacking for the development of an initial functional layer as a base for subsequent coatings on titanium surfaces. The purpose of our work was to establish a novel initial layer on Ti surfaces using phase-transited lysozyme (PTL), on which multilayer coatings can incorporate silver nanoparticles (AgNP) using chitosan (CS) and hyaluronic acid (HA) via a layer-by-layer (LbL) self-assembly technique.

Methods: In this study, the surfaces of Ti substrates were primed by dipping into a mixture of lysozyme and tris(2-carboxyethyl)phosphine (TCEP) to obtain PTL-functionalized Ti substrates. The subsequent alternating coatings of HA and chitosan loaded with AgNP onto the precursor layer of PTL were carried out via LbL self-assembly to construct multilayer coatings on Ti substrates.

Results: The results of SEM and XPS indicated that the necklace-like PTL and self-assembled multilayer were successfully immobilized on the Ti substrates. The multilayer coatings loaded with AgNP can kill planktonic and adherent bacteria, and sustained release of Ag over 14 days can prevent bacterial invasion until mucosal healing. Although the AgNP-containing structure showed some cytotoxicity, the toxicity can be reduced by controlling the Ag release rate and concentration.

Conclusions: The PTL priming method provides a promising strategy for fabricating long-term antibacterial multilayer coatings on titanium surfaces via the LbL self-assembly technique, which is effective in preventing implant-associated infections and guarantees normal early-stage wound healing.

**Key words:** phase-transited lysozyme, multilayer coatings, surface priming, hyaluronic acid, chitosan, silver nanoparticles, layer-by-layer self-assembly technique

1. **Introduction:**

Titanium (Ti) and its alloys are currently considered to be the most widely used biomaterials for dental implants because of their superior biocompatibility and excellent physicochemical properties [[1](#_ENREF_1)]. However, implant-associated infection remains one of the most perilous complications of these procedures, leading to the failure of implant surgery along with psychological trauma and economic burden [[2](#_ENREF_2)]. Recent studies have estimated that 65% of nosocomial infections are associated with biofilm, which has an enormous impact on medical therapies [[3](#_ENREF_3)]. Peri-implantitis and peri-implant mucositis are generally difficult to manage owing to the long duration of antibiotic therapy and repeated surgical procedures [[4](#_ENREF_4)]. The formation of biofilm on the surfaces of implants followed by bacterial adhesion is the primary cause of infections of the mucosa and bone adjacent to the implant [[5](#_ENREF_5)]. The process of biofilm formation involves unicellular organisms coming together to form a contiguous community encompassed in an exopolysaccharide matrix [[6](#_ENREF_6)]. Thus, the biofilm bacteria more invasive and competitive against the host defenses and presents difficulties for antibacterial treatments [[7](#_ENREF_7)]. Therefore, establishing long-term antibacterial coatings on the surfaces of titanium implants to inhibit biofilm formation is of prime importance in the prevention of implant-associated infections.

Currently, the incorporation of antibacterial drugs into the coatings of dental implant surfaces has attracted increasing attention and is considered an effective strategy to prevent bacterial adhesion and subsequent biofilm formation. Before loading antibacterial agents, it is essential to pretreat the implant surfaces using physical and chemical methods. Physical surface modification involves [[8](#_ENREF_8)] physical vapor deposition, ion beam implantation and lithographic techniques; chemical methods are considered the most popular and efficient ways to modify implant surfaces and include acid etching, peroxidation, alkali treatment, anodic oxidation, incorporation of functional molecules via covalent crosslinking, chemical vapor deposition and hydrothermal modification [[8](#_ENREF_8)]. However, these methods are inconvenient for application due to the involvement of complicated priming procedures, hazardous chemical substances and large-scale manufacturing equipment. Thus, a simple priming method for applying antibacterial coatings onto titanium surfaces is needed.

A novel phase-transited lysozyme (PTL) has recently been applied in surface functionalization. Compared with other traditional surface pretreatment techniques, priming pristine titanium surfaces with PTL is a simple, rapid, low-cost and green process for surface functionalization. The phase-transited lysozyme can be stably immobilized on a variety of substrates, regardless of the substrate type, by the formation of an amyloid-like microfiber network based on the β-sheets found in lysozyme microfibers, which enables a robust adhesion to titanium surfaces [[9](#_ENREF_9)]. This method is a one-step modification achieved by soaking the titanium surface in lysozyme transition buffer. In fact, priming with PTL places an initial layer of positive charges on the Ti surfaces for the fabrication of layer-by-layer self-assembly, on which the simple but robust immobilization of a series of functional building blocks can be accomplished through straightforward electrostatic interaction [[9](#_ENREF_9)]. Consequently, it is possible to incorporate antibacterial agents into coatings on implants simply based on PTL pretreatment combined with this layer-by-layer self-assembly technique.

Recently, the efficacy of antimicrobial agents and the corresponding antibiotic resistance to these agents are still challenges to maintaining long-term anti-infection treatments. Currently, silver nanoparticles, as strong antimicrobial agents, have attracted growing interest. In contrast with conventional antibiotics, silver nanoparticles have advantages such as strong antibacterial efficacy without the generation of drug-resistant bacterial strains, a broad antibacterial spectrum that includes antibiotic-resistant bacteria and non-cytotoxicity in moderate doses [[10](#_ENREF_10),[11](#_ENREF_11)]. Furthermore, to inhibit biofilm formation, a composite structure must be fabricated on the Ti surfaces for sustained-release of Ag and long-term antibacterial activity. Layer-by-layer (LbL) self-assembly is a well-established versatile approach that fabricates a multilayer structure on Ti surfaces by depositing alternating layers of oppositely charged polyelectrolytes [[12](#_ENREF_12)]. Multilayers composed of positively charged chitosan (CS) and negatively charged hyaluronic acid (HA) via LbL self-assembly are commonly used for drug delivery and release control due to their desirable biocompatibility [[13](#_ENREF_13),[14](#_ENREF_14)]. Moreover, chitosan also serves as a dispersant for silver nanoparticles [[15](#_ENREF_15)]. Thus, in this study, we applied the layer-by-layer (LbL) self-assembly technique to fabricate a silver nanoparticle-containing multilayer coating on PTL-primed Ti surfaces.

The aim of our study was to establish a novel initial layer on Ti surfaces with phase-transited lysozyme (PTL), based on which multilayer coatings with silver nanoparticles incorporated would be fabricated using chitosan (CS) and hyaluronic acid (HA) via the LbL self-assembly technique. We hypothesized that the silver nanoparticle-containing multilayer coating on the PTL-primed Ti surfaces would exhibit relatively long-term antibacterial efficacy, prevent biofilm formation and show favorable biocompatibility.

**2. Materials and methods**

**2.1. Materials**

Pure titanium foils of 1-mm thickness and 12-mm diameter were purchased from Baoji Noble Metal Co., Ltd. (Shanxi,China). Chitosan ([2-anino-2-deoxy-(1–4)-β-D-glucopyranosel]), with a molecular mass of 400,000 Da and deacetylation degree of 100%, was purchased from Fluka. Sodium hyaluronate (1 g) and silver nitrate (25 g, AR, ≥99.8%) were purchased from SangonCo., Ltd. (Shanghai,China). *Staphylococcus aureus* (*S. aureus*, ATCC 25923) was obtained from China General Microbiological Culture Collection Centre. Lysozyme (2 mg/mL) dissolved in HEPES buffer (pH 7.4) and tris(2-carboxyethyl)phosphine (TCEP) (20 mM) were obtained from Shaanxi Normal University.

**2.2. Specimen preparation**

The pure Ti foils were polished by SiC sandpaper of No.100, 240, 400, 600, 800, and 1000 grit in turn. Then, the foils were ultrasonically washed with acetone, ethanol and deionized water sequentially. Last, the Ti foils were sterilized in an autoclave at 120 °C for 1 h for the *in vitro* experiments.

To functionalize the surface of the Ti discs, the samples were first dipped into a mixture of lysozyme and TCEP (1:1 in volume) and then incubated in a moist environment for 2 hours. These Ti discs were then washed with ultrapure water to remove residual impurities.

**2.3. Synthesis of silver nanoparticles**

A chitosan solution was first prepared by dissolving 0.1% (w/v) chitosan in a 1% (v/v) acetic acid solution under stirring (1300 r/min). Next, silver nitrate powder was dissolved in the chitosan solution under stirring (1300 r/min) to obtain four concentrations (10, 20, 50, and 100 mM) of an AgNO_3_ solution. Subsequently, ascorbic acid (0.01 M) was added to the AgNO_3_ solution drop by drop with a pipette under constant stirring. All the above experiments were carried out at room temperature.

**2.4. Fabrication of multilayer coatings on the surface of PTL-primed Ti substrates**

After the precursor layer was established, the substrate was sequentially treated with HA (1 mg/mL in 0.2 M sodium acetate buffer), washed with sodium acetate buffer, and then covered by CS/Ag nanoparticles. HA and CS/Ag were each defined as one monolayer, and HA-CS/Ag was defined as one bilayer. These discs were denoted by Ti-PTL-HA-CS/Ag and were one typical cycle of multilayer construction. The immersion cycle was repeated three times until the desired multilayer coating was obtained (HA-CS/Ag-HA-CS/Ag-HA-CS/Ag). Finally, these samples were stored in a constant humidity chamber at 50±5% relative humidity before follow-up experiments.

**2.5. Release of silver incorporated into the composite in vitro**

The amount of silver released from the composite samples was monitored in phosphate buffered saline (PBS). The samples were immersed in 10 ml of PBS for 1 day in the dark, taken out, and then immersed again in 10 ml of fresh PBS. At different sampling intervals (1, 4, 7and 14 days), the supernatant was sampled for analysis. The PBS supernatant containing released Ag was analyzed by inductively coupled plasma atomic emission spectrometry (ICP-AES, Varian 725-ES, US).

**2.6. Surface characterization**

X-ray photoelectron spectroscopy (XPS, AXIS His, Kratos Analytical Ltd., UK) was used to identify the chemical constituents of pristine and variously modified Ti surfaces.

The contact angles of deionized water on the pristine and modified Ti surfaces were measured by the sessile drop method in a goniometer equipped with the drop-shape analysis system (JC2000D1, Micaren, China) at room temperature. Each Ti disc was measured three times to calculate the mean values of the contact angles.

The surface morphology of the pristine and decorated Ti was characterized by field-emission scanning electron microscopy (FE-SEM, JSM-5600LV, JEOL, Japan) with a beam voltage of 15kV. All the samples were sputter-coated with gold before SEM observation except for the Ti discs modified with CS/Ag nanoparticles. The chemical composition of the surface of the CS/AgNP discs was identified by energy dispersive X-ray detector (EDX, Japan).

The size and morphology of the Ag nanoparticles was observed by transmission electron microscopy (TEM, Philips CM20).

**2.7. Antibacterial assay**

*Staphylococcus aureus* (*S. aureus*, ATCC 25923) was cultivated in a beef extract-peptone (BEP) medium. After an overnight culture at 37°C, the bacterial suspension was adjusted to a concentration of 10^5^ CFU/ml for the antibacterial assay. Ti discs were put into sterilized 24-well plates filled with a bacterial suspension (1 ml per well) and cultured at 37°C in an incubator containing 5% CO_2_. At different intervals (1, 3, 5, 7 and 14 days), the bacterial suspension was sampled, and the viable planktonic bacteria were counted using serial dilutions and the spread plate method. Next, the Ti discs were taken out, gently rinsed with PBS to eliminate non-attached bacteria and then underwent ultrasonic treatment at 40 W for 5 min in a new 24-well plate filled with 1 ml of BEP per well, followed by sampling the bacterial suspension to count the viable bacteria adhered to the Ti discs. During the incubation period, the former medium was replaced by a new culture medium inoculated with bacteria every day.

The antibacterial activity rates of samples against planktonic bacteria and adhered bacteria were determined by the following formula: R= (B-A)/B×100%. A is the number of viable bacteria in the culture medium with a modified/pristine Ti disc or on a modified Ti disc. B is the number of viable bacteria in the culture medium without a Ti disc or on a pristine Ti disc.

Fluorescence staining was used to characterize the viability of adherent bacteria on the samples. *S. aureus* was seeded on the surfaces of the Ti discs in a 24-well plate, as with the incubation, for 7 days, as previously described. The bacterial medium was refreshed daily, and after 7 days, the samples were rinsed with PBS to remove non-adherent bacteria. Then, the bacterial cells were stained with acridine orange and ethidium bromide for 15 min in the dark before observation by confocal laser scanning microscopy (CLSM) (TCS SP5, Leica, Germany).

**2.8. Cell culture**

MC3T3-E1 murine preosteoblasts (Type Culture Collection of the Chinese Academy of Sciences, Shanghai, China) were used for cytotoxicity tests. Cells were cultured in DMEM medium (Gibco, Carlsbad, CA) containing 10% fetal bovine serum (FBS) (Gibco) and 3% penicillin/streptomycin (Gibco) at 37°C in a humidified atmosphere of 5% CO_2_.

**2.9. Lactate dehydrogenase activity assay**

The cytotoxicity of AgNP to MC3T3 cells can be assessed by the activity of lactate dehydrogenase (LDH, Sigma-Aldrich) released by the cells in the culture media. After incubation for 1 and 4 days, the culture media were sampled and centrifuged, and then, the supernatant was used for the LDH activity assay. LDH activity was determined by the absorbance value of optical density (OD) at a 450-nm wavelength according to the manufacturer’s instructions.

**2.10. Alkaline phosphatase activity**

One milliliter of the MC3T3 cell suspension was seeded on each specimen in a 24-well plate at a density of 1×10^5^ cells per well. The cells were cultured for 7 days, then washed with PBS and lysed in 0.1 vol% Triton X-100 through the standard freeze-thaw cycles. The alkaline phosphatase (ALP) activity in the cell suspension was determined by the absorbance value at 520 nm. The ALP activity was normalized to the total protein content, which was analyzed by the MicroBCA protein assay kit.

**2.11. Cell viability assay**

To evaluate the *in vitro* cell viability of the MC3T3 cells on each sample, a cell counting kit-8 assay (CCK-8, Dojindo, Japan) was performed according to the manufacturer’s instruction. In brief, after incubation for 3 days, the cells were collected and centrifuged before they were placed in a 96-well plate with 100 μl fresh medium per well. Next, a 25-μl CCK-8 solution was added to each well of the 96-well plate and kept at 37°C and 5% CO_2_. After two hours, the production of formazan by viable cells was assessed through the absorbance value of supernatant optical density (OD), which was measured with a microplate reader (model 680, Bio-Rad, CA) at a 450-nm wavelength.

**2.12. Statistical analysis**

Each test was repeated three times, and the results were expressed as the means ± standard deviations. The data were assessed statistically using one-way ANOVA and a Student-Newman-Keuls (SNK) post hoc test. P < 0.05 was considered significant, and p < 0.01 was considered highly significant.

**3. Results**

**3.1. Surface characterization**

The chemical constituents of the surface of the pristine and modified Ti discs in various stages of LBL self-assembly were analyzed by XPS. The XPS spectra of surface elemental compositions after calibrating peak positions, using C 1s as an internal reference at 284.8 eV, are shown in **Fig. 1** and **Table 1**. The wide-scan spectrum of the pristine Ti (**Fig. 1a**) shows that the chief components include C 1s, Ti 2p3 (458.5 eV), O 1s (530 eV) and N 1s (399 eV). The distinctive peaks of P 2p (132 eV) and S 2p (164 eV) originated from TCEP, and the disappearance of the Ti 2p3 peak indicates successful anchoring of PTL to the Ti substrate (**Fig. 1b)**, which is also supported by the quantitative analysis of the XPS results (**Table 1**). As shown in **Fig. 1c** and **Table 1**, Na content originated from hyaluronate, which indicates that hyaluronic acid (HA) was immobilized on the PTL-primed Ti surface. Upon the addition of CS/Ag nanoparticles (AgNP) to the layer of HA, the distinctive Ag content appears due to the Ag-containing in chitosan. **Fig. 2** shows the binding energies of the Ag 3d peak at 368.25 eV and 374.25 eV, which can be assigned to 3d5/2 and 3d3/2 of metallic Ag^0^ [[16](#_ENREF_16)], indicating that Ag primarily exists in the Ag^0^ state in the composite CS/AgNP .

The surface hydrophilicity of pristine and modified Ti discs was also investigated. As depicted in **Fig. 3**, the water contact angle on the Ti surface coated with the LbL self-assembled multilayer containing CS/AgNP is sharply decreased from 76.9°±2° to 49.3°±3°. The surface of the pristine Ti discs was more hydrophobic, while the surface of the modified Ti discs was hydrophilic.

SEM results (**Fig. 4a-d**) show the surface morphology of a pristine Ti disc, PTL primed Ti disc, and HA-coated PTL-Ti and multilayer of HA and CS/AgNP coated Ti disc. The SEM image (**Fig. 4b**) reveals that the PTL has necklace-like fibers with a diameter of 0.5-1 μm, which is in good agreement with a previous report [[9](#_ENREF_9)]. The EDX pattern (**Fig. 4e**) also verifies that the chemical composition of the self-assembled multilayer loaded onto the Ti surface is silver. The morphology of the composite CS/AgNP (10 mM) is exhibited in the TEM results (**Fig. 4f**). The shape of the silver nanoparticles was a relatively uniform sphere averaging approximately 30 nm in size.

**3.2. Release of Ag from Ag nanoparticle-loaded Ti discs**

As shown in **Fig. 5**, the Ag released from the samples in PBS exhibits an initial burst effect on the first day. The amount of Ag released at the different intervals (1, 4, 7, and 14 days) follows the order of CS/Ag100 > CS/Ag50 > CS/Ag20 > CS/Ag10. Initially, relatively large amounts of Ag were released into the PBS, with CS/Ag100 leaching the most, and the amount of released Ag decreased gradually with immersion time and tended to be stable after 7 days. After two weeks, the average concentration of released Ag was 0.70±0.14 μg/ml.

**3.3. Inhibition of biofilm formation**

The antimicrobial ability of CS/Ag-decorated Ti discs was investigated by fluorescence staining. **Fig. 6** shows the CLSM images of adherent bacteria on the pristine and modified Ti discs after 7 days. The Ti discs were incubated in the culture media with repeated bacterial invasion every day. The fluorescence microscopy images of CLSM showed more dead *S. aureus* cells on the surfaces of the CS/Ag samples with red color. In addition, viable bacteria with green color were observed on the surfaces of pristine Ti and PTL-primed Ti. In significant contrast, nearly no viable bacterial cells could be found on the CS/Ag-modified Ti surfaces.

The antibacterial activity rates of samples against planktonic bacteria in the medium (Rp) and adherent bacteria on the surfaces of samples (Ra) over 14 days were evaluated, as shown in **Figs. 7 and 8**, respectively. The CS/Ag samples showed Rp values of approximately 100%, significantly higher than pristine Ti during the first 4 days. At the 5^th^ day, the Rp values of the CS/Ag samples decreased gradually, and those of the CS/Ag10 samples diminished more rapidly. After 7 days, the Rp values of the CS/Ag samples, particularly the CS/Ag100 samples, were significantly higher than those of pristine Ti samples. The modified Ti surfaces with silver incorporated were effective in preventing bacterial colonization on the Ti discs for 14 days as illustrated by **Fig. 8**. Most of the CS/Ag samples showed Ra values of 100% without a significant decrease over 7 days. However, the CS/Ag10 samples exhibited a relatively sharp decrease after 14 days, reaching a value of approximately 65%. The other three CS/Ag groups still showed Ra values of approximately 90% after 14 days.

**3.4. Cytotoxicity**

The cytotoxicity results indicated by the LDH activity in the supernatants after 1 and 4 days of incubation are compared in **Fig. 9**. After the first day, neither the Ti-PTL nor the CS/Ag samples showed an obvious enhancement in LDH activity. After culturing for 4 days, the CS/Ag20 and CS/Ag50 samples exhibited slightly higher LDH activity than pristine Ti, Ti-PTL and CS/Ag10, but the difference was statistically insignificant. However, higher LDH activity was observed in the CS/Ag100 sample. The CS/Ag samples exhibited cytotoxicity with the increase in the amount of incorporated Ag.

**3.5. Cell viability**

The cell viability of each sample was evaluated by a Cell Counting Kit 8 (CCK-8). As shown in **Fig. 10**, the CS/Ag20, CS/Ag50, and CS/Ag100 samples show significant differences without the CS/Ag10 group. Moreover, CS/Ag100 samples exhibited significantly lower cell viability than the other samples.

**3.6. Alkaline phosphatase activity**

The ALP activity assay after 7 days of culturing is shown in **Fig. 11**. Compared with pristine Ti, the modified Ti discs with Ag incorporated had decreased ALP activity, particularly the CS/Ag100 samples, which exhibited dramatically lower ALP activity (20-35%).

**4. Discussion**

In this study, we tried to develop a distinctive and simple pretreatment method using phase-transited lysozyme (PTL) to modify the Ti surfaces. The initial layer of PTL on Ti surfaces would provide a base for antibacterial multilayer coatings established via a layer-by-layer self-assembly technique. Ideally, a broad spectrum of building blocks, including small and macro-molecules, biomolecules, and colloids, could be easily immobilized onto a PTL-primed substrate, regardless of substrate type [[9](#_ENREF_9)]. XPS results (**Fig. 1 b**) demonstrated that the PTL was successfully immobilized onto the surface of Ti discs. It has been suggested that PTL is an extremely stable material, and the adhesion strength of PTL is strong enough to endure ultrasonic vibration [[9](#_ENREF_9)]. The PTL immobilized on substrates remains intact in various polar and non-polar organic solvents as well as acids, bases, inorganic salts, surfactants and oxidants, with the exception of guanidine solution (GndCl) [[9](#_ENREF_9)]. The adhesion feature of PTL originates from amyloids found in the lysozyme fibers, which have commonly been used as proteinaceous underwater adhesives for bioadhesion [[9](#_ENREF_9)]. This mechanism may be ascribed to a complex sequence of events and the co-contributions of multi-scale molecular and structural amyloid bonds including osmotic pressure-driven solvent depletion force, hydrophobic interactions, physical entanglement and hydrogen bonding/electrostatic interactions [[9](#_ENREF_9)]. In addition, compared with negatively charged dopamine, which is widely applied to prime surfaces for further modification [[17](#_ENREF_17)], PTL contains polar functionalities such as amines and hydroxyls with mild positive charges over a broad pH range for robust immobilization of negatively charged functional building blocks on Ti substrates [[9](#_ENREF_9)].

The formation of biofilm around dental implants is the essential factor in the evolution and persistence of infection [[18](#_ENREF_18)]. Moreover, the surgical trauma of implantation can disturb the host defense system, which facilitates bacterial colonization [[19](#_ENREF_19)]. Thus, it is absolutely necessary to develop effective strategies to prevent implant-associated infections. Although antibacterial coatings loaded onto Ti surfaces have attracted considerable attention due to the effective inhibition of biofilm formation and relatively long-term antibacterial effect, which are crucial to protecting biomedical implants against the constant risk of infection before mucosal healing. In contrast with monolayer antibacterial coatings, multilayer coatings on a Ti surface constructed by layer-by-layer (LbL) self-assembly technique can enhance loading capacity and control the release of antibacterial agents to achieve a long-term antibacterial effect [[20](#_ENREF_20)].

In the present work, multilayer coatings on PTL-primed Ti surfaces were fabricated by alternate adsorptions of polyanions (hyaluronic acid) and polycations (chitosan) through electrostatic interaction based on an LbL self-assembly technique. The whole process is shown in **Fig. 12**. XPS results indicated that the self-assembled multilayer of chitosan and hyaluronic acid was successfully coated onto the surface of Ti discs (**Fig. 1d and Table 1**). The chitosan layers were loaded with Ag nanoparticles as antibacterial agents. In present study, the Ag nanoparticles were synthesized in a chitosan medium with the addition of the reducing agent VC. The linear macromolecules of chitosan will form tridimensional gridding structures, providing host spaces for silver ions [[21](#_ENREF_21)]. Thus, in the chitosan medium, the synthesis of Ag nanoparticles with controlled size, morphology and dispersity can be accomplished when these silver ions are reduced in situ to form nanoparticles as shown in **Fig. 4e**, producing complexes of chitosan and Ag nanoparticles (CS/AgNP). Moreover, the complexes of CS/AgNP still remained positively charged and thus could be directly adsorbed onto the layer of HA (**Fig. 4d and Fig. 1d**). This one-step fabrication of complexes of CS/AgNP is a simple and convenient method to load an antibacterial coating onto Ti surfaces via the LbL self-assembly technique.

In the present work, the multilayer coatings loaded with various concentrations of AgNP showed effective antibacterial activity over a 14-day period (**Fig. 7 and 8**). The results suggested that the antibacterial activity is enhanced with increasing concentrations of AgNP. The planktonic bacteria in the medium and the adherent bacteria on the surfaces of samples were almost eradicated by AgNP released from the self-assembled multilayer during the first 5 days, thus reducing the bacterial colonization of the surfaces of samples. The antimicrobial effect could be ascribed to the release of Ag^+^ from AgNP, which has the ability to inhibit bacterial DNA replication, interrupt bacteria cellular processes and induce reactive oxygen species (ROS) [[22](#_ENREF_22),[23](#_ENREF_23)]. ROS can increase the permeability of the bacterial membrane, causing bacteria to be more susceptible to antibacterial agents. It is worth mentioning that the bacterial suspension in the antibacterial assay was adjusted to the concentration of 10^5^CFU/ml and changed every 24 h to ensure that the samples were subjected to a strong bacterial attack. The abovementioned conditions are harsher than the physical conditions *in vivo*, and thus, the efficacy of the antibacterial multilayer coatings is expected to be sustainable for a longer period of time under physical conditions [[16](#_ENREF_16)]. In addition, unlike the relative hydrophobicity of pristine Ti, the hydrophilicity of Ti surfaces coated with a multilayer of HA and CS/AgNP (**Fig. 3**) contributes to the reduction in bacterial cells [[20](#_ENREF_20)]. The results of the antibacterial activity rate against adherent bacteria on the surfaces of samples demonstrate that the self-assembled multilayer of chitosan and hyaluronic acid loaded with AgNP can effectively inhibit biofilm formation on Ti surfaces over 14 days.

An adequate period of time and a suitable profile of drug release at an effective antibacterial concentration are necessary to inhibit biofilm formation before wound healing. Initially, a large amount of Ag was released from the self-assembled multilayer into the PBS, which was attributed to the outermost layer of chitosan loaded with AgNP, but Ag release gradually decreased with the increase in immersion time (**Fig. 5**). The Ag release profile over 14 days observed in our study is advisable because a surgical wound will heal within 10-14 days, after which the constant release of Ag is not recommended due to cytotoxicity to the host cells. The initial burst of Ag release inhibits the immediate colonization of bacteria on the Ti surfaces after implant surgery. Next, sustained release of Ag is required to resist bacterial invasion from the outer edges of the wound until mucosal healing. In our design of the self-assembled multilayer, the hyaluronic acid (HA) layer played a role in retarding the release of Ag nanoparticles from the lower layer of chitosan. This mechanism is similar to that of the controlled release of minocycline from alginate-coated chitosan microspheres [[20](#_ENREF_20)]. In this study, the four concentration levels of AgNP were able to effectively kill planktonic and adherent bacteria over 14 days; the cytotoxicity of the four levels was also investigated to find a proper concentration for ensuring normal mucosal healing.

Concerning the toxicity of silver, it is generally considered that Ag is biocompatible at low concentrations and cytotoxic to host cells at high doses [[16](#_ENREF_16),[24](#_ENREF_24)]. This is related to a large release of Ag ions due to the high surface energy of smaller AgNP [[22](#_ENREF_22)]. For biomedical implant applications, smaller Ag particle size does not always lead to better performance. In our work, in comparison to pristine Ti, the multilayer coatings with AgNP incorporated (10, 20, 50, 100 mM) still showed cytotoxicity against MC3T3 cells to a certain extent, as demonstrated by the LDH, ALP activity and cell viability assays (**Fig. 9-11**). However, the samples of CS/Ag10 with the lowest concentration of AgNP showed better biocompatibility than other groups during the first 4 days according to the LDH activity assay results (**Fig. 9**). The cytotoxicity of the samples may be attributed to the leaching of silver ions and their accumulation in the culture medium during the incubation period [[16](#_ENREF_16),[22](#_ENREF_22),[25](#_ENREF_25)]. After 3 days of culturing without changing the culture medium, the cumulative concentration of silver ions led to cytotoxicity at day 4. However, recent studies indicate that biomaterials containing a proper amount of silver are biocompatible with osteoblasts [[10](#_ENREF_10),[11](#_ENREF_11),[26](#_ENREF_26),[27](#_ENREF_27)]. As a result, it is desirable that the bacteria be killed without inducing cell cytotoxicity at a properly low concentration of silver ions. Ti surfaces coated with a HA-CS/AgNP multilayer via PTL-priming and the LbL self-assembly technique can exhibit good biocompatibility by controlling the release of silver. Therefore, the antibacterial and biocompatible surfaces of titanium modified with the HA-CS/AgNP multilayer have a property that prevents post-operation infection in the early stage of implantation, which will be further investigated through future *in vivo* experiments.

In conclusion, in contrast to other well-established methods, our surface-priming strategy provides an extremely facile, green and powerful approach to preparing Ti surfaces by using PTL coating, without time-consuming chemical syntheses and costly processing [[9](#_ENREF_9)]. Therefore, the initial layer of PTL holds great potential for the fabrication of multilayers loaded with antibacterial agents, osteogenic growth factors, cytokines and/or other functional components on Ti surfaces via the LbL self-assembly technique, which could prevent implant-associated infection and facilitate osseointegration in the early stage of implantation.

**Acknowledgments**

This work was jointly supported by the National Natural Science Foundation of China (Grant No: 31470920), National Natural Science Foundation of China (Grant No: 21374057, 51303100) and Tianjin Research Program of Application Foundation and Advanced Technology (Grant No: 12JCZDJC22700).

**References**

1. Neoh KG, Hu X, Zheng D, Kang ET (2012) Balancing osteoblast functions and bacterial adhesion on functionalized titanium surfaces. Biomaterials 33: 2813-2822.

2. Darouiche RO (2004) Treatment of infections associated with surgical implants. N Engl J Med 350: 1422-1429.

3. Mah TF, O'Toole GA (2001) Mechanisms of biofilm resistance to antimicrobial agents. Trends Microbiol 9: 34-39.

4. Camargo IB, Van Sickels JE (2015) Surgical Complications After Implant Placement. Dental Clinics of North America 59: 57-72.

5. Koseki H, Yonekura A, Shida T, Yoda I, Horiuchi H, et al. (2014) Early staphylococcal biofilm formation on solid orthopaedic implant materials: in vitro study. PLoS One 9: e107588.

6. Limoli DH, Jones CJ, Wozniak DJ (2015) Bacterial Extracellular Polysaccharides in Biofilm Formation and Function. Microbiol Spectr 3.

7. Esposito M, Hirsch JM, Lekholm U, Thomsen P (1998) Biological factors contributing to failures of osseointegrated oral implants. (I). Success criteria and epidemiology. Eur J Oral Sci 106: 527-551.

8. Yeo IS (2014) Reality of dental implant surface modification: a short literature review. Open Biomed Eng J 8: 114-119.

9. Wu Z, Yang P (2015) Simple Multipurpose Surface Functionalization by Phase Transited Protein Adhesion. Advanced Materials Interfaces 2: n/a-n/a.

10. Hardes J, Ahrens H, Gebert C, Streitbuerger A, Buerger H, et al. (2007) Lack of toxicological side-effects in silver-coated megaprostheses in humans. Biomaterials 28: 2869-2875.

11. Alt V, Bechert T, Steinrucke P, Wagener M, Seidel P, et al. (2004) An in vitro assessment of the antibacterial properties and cytotoxicity of nanoparticulate silver bone cement. Biomaterials 25: 4383-4391.

12. Huang R, Li W, Lv X, Lei Z, Bian Y, et al. (2015) Biomimetic LBL structured nanofibrous matrices assembled by chitosan/collagen for promoting wound healing. Biomaterials 53: 58-75.

13. Song W, Song X, Yang C, Gao S, Klausen LH, et al. (2015) Chitosan/siRNA functionalized titanium surface via a layer-by-layer approach for in vitro sustained gene silencing and osteogenic promotion. Int J Nanomedicine 10: 2335-2346.

14. Jeon S, Yoo CY, Park SN (2015) Improved stability and skin permeability of sodium hyaluronate-chitosan multilayered liposomes by Layer-by-Layer electrostatic deposition for quercetin delivery. Colloids Surf B Biointerfaces 129: 7-14.

15. Sanpui P, Murugadoss A, Prasad PV, Ghosh SS, Chattopadhyay A (2008) The antibacterial properties of a novel chitosan-Ag-nanoparticle composite. Int J Food Microbiol 124: 142-146.

16. Zhao L, Wang H, Huo K, Cui L, Zhang W, et al. (2011) Antibacterial nano-structured titania coating incorporated with silver nanoparticles. Biomaterials 32: 5706-5716.

17. He S, Zhou P, Wang L, Xiong X, Zhang Y, et al. (2014) Antibiotic-decorated titanium with enhanced antibacterial activity through adhesive polydopamine for dental/bone implant. J R Soc Interface 11: 20140169.

18. Noda K, Arakawa H, Kimura-Ono A, Yamazaki S, Hara ES, et al. (2015) A longitudinal retrospective study of the analysis of the risk factors of implant failure by the application of generalized estimating equations. J Prosthodont Res 59: 178-184.

19. Zhao L, Chu PK, Zhang Y, Wu Z (2009) Antibacterial coatings on titanium implants. J Biomed Mater Res B Appl Biomater 91: 470-480.

20. Lv H, Chen Z, Yang X, Cen L, Zhang X, et al. (2014) Layer-by-layer self-assembly of minocycline-loaded chitosan/alginate multilayer on titanium substrates to inhibit biofilm formation. J Dent 42: 1464-1472.

21. Xu Z, Peng Y, Wantai Y, Jinchun C (2008) The bio-inspired approach to controllable biomimetic synthesis of silver nanoparticles in organic matrix of chitosan and silver-binding peptide (NPSSLFRYLPSD). Materials Science and Engineering: C 28: 237-242.

22. Zhao Y, Xing Q, Janjanam J, He K, Long F, et al. (2014) Facile electrochemical synthesis of antimicrobial TiO(2) nanotube arrays. Int J Nanomedicine 9: 5177-5187.

23. Ahamed M, Alsalhi MS, Siddiqui MK (2010) Silver nanoparticle applications and human health. Clin Chim Acta 411: 1841-1848.

24. Zhang X, Li Z, Yuan X, Cui Z, Bao H, et al. (2013) Cytotoxicity and antibacterial property of titanium alloy coated with silver nanoparticle-containing polyelectrolyte multilayer. Mater Sci Eng C Mater Biol Appl 33: 2816-2820.

25. Qin H, Cao H, Zhao Y, Zhu C, Cheng T, et al. (2014) In vitro and in vivo anti-biofilm effects of silver nanoparticles immobilized on titanium. Biomaterials 35: 9114-9125.

26. Agarwal A, Weis TL, Schurr MJ, Faith NG, Czuprynski CJ, et al. (2010) Surfaces modified with nanometer-thick silver-impregnated polymeric films that kill bacteria but support growth of mammalian cells. Biomaterials 31: 680-690.

27. Qiao S, Cao H, Zhao X, Lo H, Zhuang L, et al. (2015) Ag-plasma modification enhances bone apposition around titanium dental implants: an animal study in Labrador dogs. Int J Nanomedicine 10: 653-664.

|  | C% | O% | N% | Ti% | Na% | S% | Ag% | P% |
| --- | --- | --- | --- | --- | --- | --- | --- | --- |
| Ti | 47.2±0.2 | 38.2±0.3 | 4.4±0.1 | 10.2±0.03 | 0 | 0 | 0 | 0 |
| Ti-PTL | 62.7±0.3 | 18.3±0.2 | 18.2±0.1 | 0 | 0 | 0.7±0.1 | 0 | 0.1±0.03 |
| Ti-PTL-HA | 60.2±0.4 | 28.8±0.3 | 9.0±0.2 | 0 | 2.0±0.02 | 0 | 0 | 0 |
| Ti-PTL-HA-CS/Ag | 49.2±0.2 | 26.5±0.1 | 10.6±0.3 | 0 | 0 | 0 | 13.8±0.2 | 0 |
| Ti-PTL-HA-CS/Ag-HA | 46.8±0.1 | 28.2±0.1 | 11.9±0.2 | 0 | 0.2 | 0 | 12.9±0.2 | 0 |

**Table 1** **Elemental composition at the surface of various Ti discs with different treatments as determined by XPS.**

**Fig. 1** **XPS wide-scan spectra of** (a) pristine Ti, (b) PTL treated Ti (Ti-PTL), (c) Ti-PTL-HA, (d) Ti-PTL-HA-CS/Ag, (e) Ti-PTL-HA-CS/Ag –HA.

**Fig. 2 High-resolution XPS spectra of Ag3d on surface of Ti-PTL-HA-CS/Ag**.

**Fig. 3** **Images of contact angle on various Ti discs**: (a) pristine Ti, (b) Ti-PTL, (c) Ti-PTL-HA, (d) LbL-CS/Ag, (e) Statistical analysis of the contact angle measurements.

**Fig. 4** **SEM images of surface morphology of Ti discs**: (a) pristine Ti, (b) Ti-PTL, (c) Ti-PTL-HA, (d) Ti-PTL-HA-CS/Ag10. (e) TEM image of CS/Ag (10 mM). (f) EDX image of the sample of Ti-PTL-HA-CS/Ag.

**Fig. 5** **Non-cumulative silver release profile in PBS**.

**Fig. 6** **CLSM fluorescence microscopy images of** (a) pristine Ti, (b) Ti-PTL, (c) LbL-CS/Ag10, (d) LbL-CS/Ag20, (e) LbL-CS/Ag50, (f) LbL-CS/Ag100 showing viability of the bacteria on samples after 7 days.

**Fig. 7** **Antibacterial activity rates against planktonic bacteria in medium (Rp)**. The antibacterial assay data are expressed as the means±standard deviations (n=3).

One-way ANOVA followed by SNK post hoc test is utilized to determine the level of significance. *p<0.05 and **p<0.01.

**Fig. 8** **Antibacterial activity rates against adherent bacteria on the specimens (Ra)**.

**Fig. 9 LDH activity in medium after culturing for 1 and 4 days on the specimens.**

**Fig. 10** **Cell viability of MC3T3 on the specimens after culturing for 3 days as determined by CCK-8 assay**.

**Fig. 11** **ALP activity of MC3T3 on the specimens after incubation for 7 days.**

**Fig. 12** **Schematic diagram:** the process of fabricating the multilayer coatings on the PTL-primed Ti surface and the mechanism of antibacterial ability of this sample under aqueous conditions. As water contacts the surface, the nanoparticles are oxidized to release Ag^+^.
